# Coronary Artery Dimensions in Endurance Athletes by Computed Tomography Angiography: A Quantitative Analysis

**DOI:** 10.3390/jcdd8110141

**Published:** 2021-10-28

**Authors:** Christian Langer, Fabian Barbieri, Fabian Plank, Christoph Beyer, Benito Baldauf, Guy Friedrich, Gerlig Widmann, Anna Luger, Agne Adukauskaite, Markus Reinthaler, Wolfgang Dichtl, Shunichi Homma, Gudrun Maria Feuchtner

**Affiliations:** 1 Department of Radiology, Medical University of Innsbruck, 6020 Innsbruck, Austria; christian.langer@tirol-kliniken.at (C.L.); Gerlig.Widmann@i-med.ac.at (G.W.); Anna.Luger@i-med.ac.at (A.L.); 2 Department of Internal Medicine III, Medical University of Innsbruck, 6020 Innsbruck, Austria; Fabian.Barbieri@i-med.ac.at (F.B.); Fabian.Plank@i-med.ac.at (F.P.); Christoph.Beyer@i-med.ac.at (C.B.); Guy.Friedrich@tirol-kliniken.at (G.F.); Agne.Adukauskaite@tirol-kliniken.at (A.A.); Wolfgang.Dichtl@tirol-kliniken.at (W.D.); 3 Department of Cardiology, Charité-Universitätsmedizin Berlin, Corporate Member of Freie Universität Berlin, Humboldt-Universität zu Berlin, and Berlin Institute of Health, Campus Benjamin Franklin, 12203 Berlin, Germany; Markus.Reinthaler@charite.de; 4 Medical Faculty, Christian-Albrechts University, 24118 Kiel, Germany; sportmedic@gmail.com; 5 Institute of Active Polymers and Berlin-Brandenburg Center for Regenerative Therapies, Helmholtz-Zentrum Hereon, 14513 Teltow, Germany; 6 Division of Cardiology, Department of Medicine, Columbia University Medical Center, New York, NY 10032, USA; sh23@cumc.columbia.edu

**Keywords:** athletes, coronary arteries, imaging, computed tomography

## Abstract

(1) Background: The athlete’s heart may develop permanent vessel enlargement. The purpose of our study was to define normal values for coronary artery dimensions of endurance athletes by coronary computed tomography angiography (CTA). (2) Methods: Ninety-eight individuals (56.2 ± 11 years) were included into this retrospective matched case-controlled-study. Endurance athletes had regular training volumes of ≥1 h per unit, ≥3–7 times per week (either cycling, running or mountain-endurance). Athletes were matched for age and gender with sedentary controls using propensity score. Quantitative CTA analysis included coronary vessel dimensions (two diameters and area) of the LM, LAD, CX and RCA for all AHA-16-segments. (3) Results: Proximal LAD area and diameter (*p* = 0.019); proximal/mid CX (diameter and area; *p* = 0.026 and *p* = 0.018/*p* = 0.008 and *p* = 0.009); mid RCA diameter and area; and proximal RCA diameter were significantly larger in endurance athletes (*p* < 0.05). The left main area (*p* = 0.708) and diameter (*p* = 0.809) as well as the mid LAD and distal segments were not different. We present the histograms and data for normal values ±1 and ± 2 SD. (4) Conclusions: Endurance athletes have larger proximal LAD, proximal/mid CX and RCA vessel dimensions, while LM and distal segments are similar. Hence, dilated coronary arteries in endurance athletes (“*Athlete’s arteries*”) have to be distinguished from diffuse ectatic segments developing during Kawasaki disease or multisystemic inflammation syndrome after COVID-19.

## 1. Introduction

The athlete’s heart is characterized by global chamber enlargement [1]. Due to prolonged vasodilatation during high exercise volumes, vascular adaption mechanisms triggered by a complex triad of growth factors [2] and arterial remodeling [3] may result in permanent coronary vessel dilatation. However, there is only limited data available [4,5,6], mostly assessing vasodilator capacity during adenosine, and in very small cohorts only. No study has yet defined normal values for coronary vessel dimensions at rest, using the most accurate imaging technology, quantitative coronary computed tomography angiography (CTA).

Kawasaki disease (KD) and the more recently emerging multisystemic inflammatory syndrome after COVID-19 [7,8,9] may cause either diffuse or focal coronary ectasia. Both are indications for coronary CTA and therefore represent important differential diagnosis in clinical practice. Rheumatic, autoimmune or other systemic diseases triggering vasculitis (i.e., IgG-4 mediated, eosinophilic granulomatous polyangiitis (EGPA)) may also involve coronary arteries and cause ectatic segments. Coronary computed tomography angiography (CTA) is advocated by the current European Society of Cardiology (ESC) guidelines [10] for examination of athletes, for example in those with a new onset of high-grade arrhythmia or chest pain.

Therefore, being aware of the normal dimensions of an “*athlete’s artery*” in a routine coronary CTA is of importance in order to establish differential diagnosis in daily clinical practice.

Accordingly, the purpose of our study was to define coronary artery vessel size (diameter, area and volumes) by quantitative coronary CTA in high-performing endurance athletes, as compared to sedentary controls.

## 2. Materials and Methods

### 2.1. Study Population

100 patients who were examined with coronary CTA for clinical indications between 01/2015 and 05/2017 were recruited. The patients had low-to-intermediate pre-test probability of coronary heart disease, and/or another prior test finding (e.g., ECG-treadmill, resting ECG or echocardiography) requiring imaging work-up. Institutional review board (IRB) waived written informed consent for this retrospective study. Endurance athletes were matched with sedentary controls for age and gender using propensity score (1:1). All were Caucasians.

### 2.2. Inclusion Criteria

*Endurance athletes* were characterized by a regular exercise volume of a minimum (≥3–7x/week) with ≥1 h duration/unit, for a minimum of 1 year. The patients filled a questionnaire prior to CTA exam, including the frequency of training per week stratified as: low (1–2x), moderate (3–5x) and high (5–7x). The time per unit was classified as: low (<30 min), moderate (≥1 h) or high (>3 h). The years of training were recorded. Endurance athletes were performing cycling (road or mountain bike), running, cross-country skiing and/or other mountain endurance sport (e.g., alpine hiking) and/or ski touring, or swimming for a minimum of >=1 year regularly and continuously. Persons who reported low-intensity running activities such as “jogging”, “Nordic walking” or “mountain walking or hiking” were excluded. Individuals who reported “irregular” activities (running, biking and others) were excluded.

*Sedentary controls* were asked prior to CTA if they performed any physical activity (including regular walking) or not and were defined as “sedentary” only if stating a clear “no”. Conventional cardiovascular (CV) risk factors were collected: arterial hypertension (systolic blood pressure > 140 mmHg or diastolic blood pressure > 90 mmHg), dyslipidemia (c-LDL > 116 mg/dl), family history (myocardial infarct or sudden cardiac death in an immediate male relative < 55 y or female < 65 y), smoking (current or quit within the last 6 months), body mass index (BMI) and diabetes.

Exclusion Criteria were previous percutaneous coronary intervention (PCI), coronary artery bypass grafting (CABG), heart valve surgery or valvular disease ≥ 2, renal dysfunction (serum glomerular filtration rate <45 mL/min/1.73 m^2^), pregnancy, acute instable chest pain, acute coronary syndrome, positive troponin T suggestive for myocardial injury, signs of systemic inflammation such as fever, c-reactive protein (CRP) elevation, systemic inflammatory diseases (e.g., rheumatic, autoimmune disease, vasculitis), prior or current Kawasaki disease and human immune deficiency virus (HIV) infection.

Computed Tomography Calcium Score and Coronary Computed Tomography Angiography. A non-contrast ECG-gated coronary artery calcium score (CACS) with standardized scan parameters (detector collimation 64 × 1.5 mm; 120 kV) was performed. The Agatston Score (AU) was calculated. Then, coronary CTA was performed with a 128-slice dual source CTA (*Definition FLASH, Siemens)* with a detector collimation of 2 × 64 × 0.6 mm and a z-flying spot and a rotation time of 0.28 s, respectively. Prospective ECG-triggering was used in regular heart rates < 65 bpm (diastolic padding, 70% of RR-interval) and retrospective ECG-gating in heart rates > 65 bpm and irregular rates. An iodine contrast agent (*Iopromide, Ultravist 370™)* was injected intravenously (flow rate 4–6 mL/s+40 cc saline), triggered into arterial phase (bolus tracking; 100 HU threshold; ascending aorta). Contrast volume was tailored to the individual patient characteristics (body weight and scan time). Volume was on average 70–80 mL (range, 65–120 mL). Axial images were reconstructed with 0.75 mm slice width (increment 0.4/medium-smooth kernel B26f and iterative reconstruction SAFIRE 3) during best diastolic and systolic phase.

### 2.3. Coronary CTA Image Analysis

**Figure 1:**
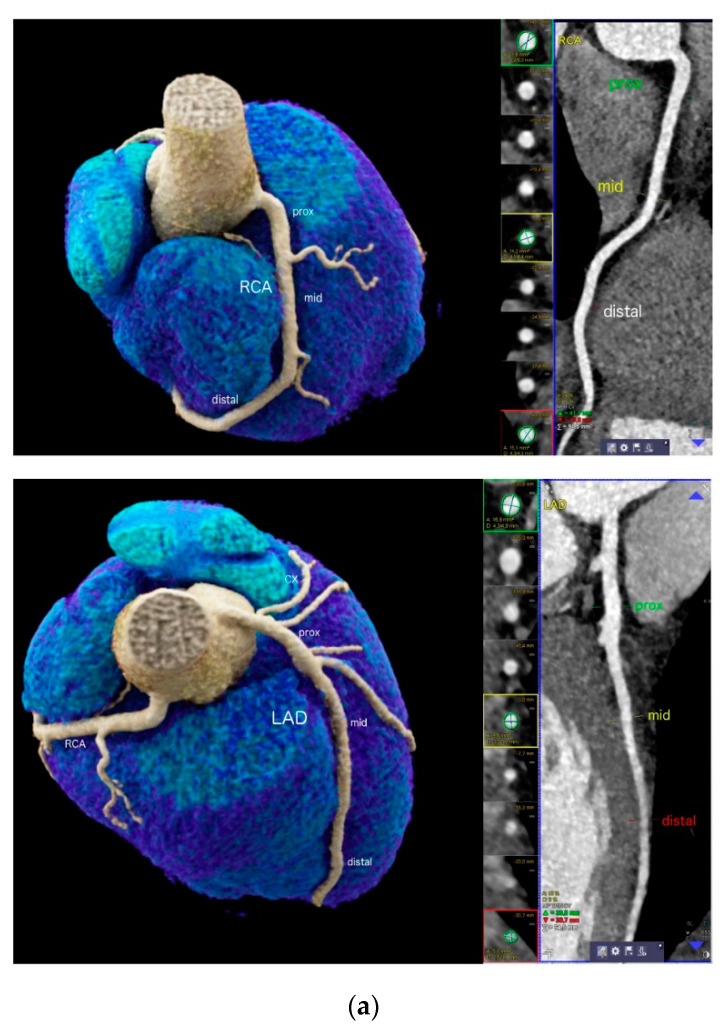

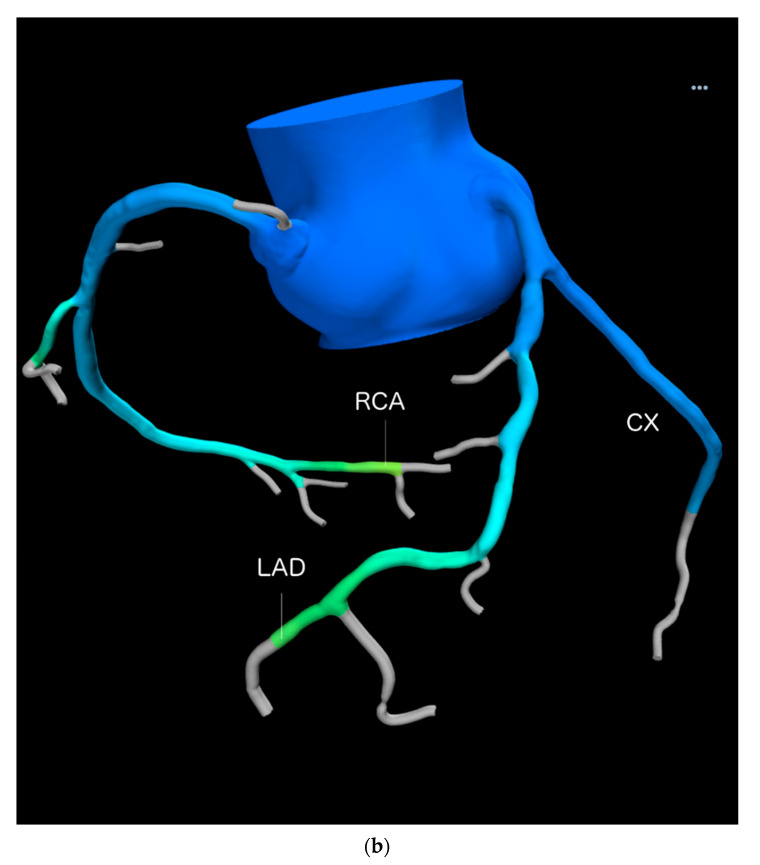
(**a**) 22-year-old professional male endurance athlete (swimmer) with regular high training volumes and a new onset of high-grade arrhythmia. Enlarged proximal but smaller mid and distal RCA (upper) and LAD (lower panel) segments (“*athlete’s artery*”). Quantitative CTA (right) included sizing of RCA and LAD dimensions at 3 sites (proximal, mid and distal) using curved multiplanar reformations (cMPR). Both vessel area (mm^2^) and 2 diameters (mm) were taken. Left panels show volume rendering technique (VRT). CX = circumflex artery. (**b**) 3D Quantification of vessel volumes (mm^3^) for the RCA, LAD and CX by using a computational fluid modelling (CFD) software (Heartflow, Inc. Redwood, CA, USA) in another patient.

Curved multiplanar reformations (cMPR) and oblique interactive MPR of all vessels using 3D post-processing software (*SyngoVia^TM^, Siemens*) were generated:

- Vessel size was measured using curved multiplanar reformations (cMPR) along a centerline on a cross-sectional perpendicular image, by a digital sizing tool in 2 dimensions (diameter 1 and 2) and effective vessel area, for each coronary segment using (AHA-modified-16-segment classification) [11] (Figure 1a). Vessel lumen was extracted automatically, and manual adjustments were made in case of outer lumen contour inaccuracies. The measurements were taken at a normal luminal site without coronary plaque, and the largest diameter was calculated. Segments with image quality limitations (e.g., artifacts, severe calcification) were excluded.
- Total vessel volume for LAD, CX and RCA was quantified using computational fluid modeling (CFD) from the end-diastolic dataset consisting of axial images according to a standardized regimen [12] (Heartflow Inc., Redwood City, California, USA). The vessel volumes were segmented using a 3-dimensional volume segmentation tool, from left ventricular mass. Total vessel lumen, volumes of each artery (left anterior descending (LAD)), right coronary artery (RCA), circumflex (CX) and myocardial mass were calculated (Figure 1b).
- Coronary stenosis above > 50% was scored visually.

CTA image analysis was performed by one experienced radiologist (>5 years’ experience) and a supervisor (>10 years’ experience), in consensus.

### 2.4. Statistical Analysis

Quantitative variables are expressed as mean ± standard deviation (SD), categorical variables reported as absolute values and percentages. Normal distribution was assessed with the Kolmogorov−Smirnov test to choose adequate parametric test, and with the D’Agostino and Pearson omnibus K2 test as well as inspection of histograms to select an adequate fitting method of Gaussian distribution curve. Mean differences between continuous data were tested by using the appropriate tests according to their distribution (t-test for normally distributed and Mann−Whitney U for non-normally distributed data). Differences in categorical data between groups were determined with chi-square or Fisher’s exact test (if n ≤ 5 per group). Gaussian fitting curves were calculated by using non-linear regression-fitting models with adequately binned histograms. Fitting method (least square fit and robust fit) was selected by distribution and convergence of fitting curves. The Spearman correlation coefficient was applied for correlation of vessel size (mean diameter and area) and total vessel volume of the LAD, and Pearson’s was applied for the RCA. Statistical analysis was performed by using IBM SSPS™ software (V24.0, IBM Corporation, Armonk, NY, USA) and GraphPad PRISM, version 5 (GraphPad Software, Inc., La Jolla, CA, USA). Graphics were designed with GraphPad PRISM. A *p*-value of less than 0.05 was considered significant.

## 3. Results

Table 1 shows the study population. Of 100 patients, two were excluded (*n* = 1 with prior CABG surgery, and *n* = 1 due to severe vessel wall calcifications). Finally, 51 athletes were compared with 47 sedentary controls. There was no difference in age, gender and the major cardiovascular risk factors between athletes and controls. Body mass index was slightly lower in the athletes. Body surface area (BSA) was not different. The prevalence of >50% coronary stenosis was low (15.7%) in athletes and not different to inactive controls. The Coronary Artery Calcium score (CACS) was not different (*p* = 0.067) as well, respectively. Twenty-one (41.2%) endurance athletes were participating in competitions. The majority (71%) were training regularly for more than 5 years. One quarter (25%) was mainly running, 36% performed mountain-endurance sport (e.g., professional alpine mountaineering and/or ski-touring, and mountain biking), 33% were mainly cycling (+other type of endurance sports, e.g., triathlon) and 6% performed other types (e.g., swimming). The exercise volume in metabolic equivalent of training (MET) was mean 8.34 ± 3.46 SD minutes/week.

Table 2 shows the coronary vessel dimensions (mean diameter and area) for all coronary segments in athletes compared to inactive controls. Out of 896 segments, 38 (4.2%) were further excluded due to artifacts (e.g., motion blurring) or a high atherosclerotic plaque load along the entire segment, not permitting accurate measurements. Measurements of the RCA included right-dominant and intermediate supply types only, whereas all left coronary artery (LCA) segments were measured in the entire cohort. Proximal left anterior descending (LAD), proximal/mid circumflex (CX) and RCA vessel sizes were significantly larger in endurance athletes for both area and the mean diameter (of 2 diameters) than in sedentary controls. Left main (LM), mid LAD and all distal segments were not different. Total vessel volumes were slightly higher but not statistically different. Myocardial mass was higher in athletes (*p* = 0.04).

Figure 1a shows a 22-year-old male professional swimmer, highlighting the enlarged proximal segments.

**Figure 2:**
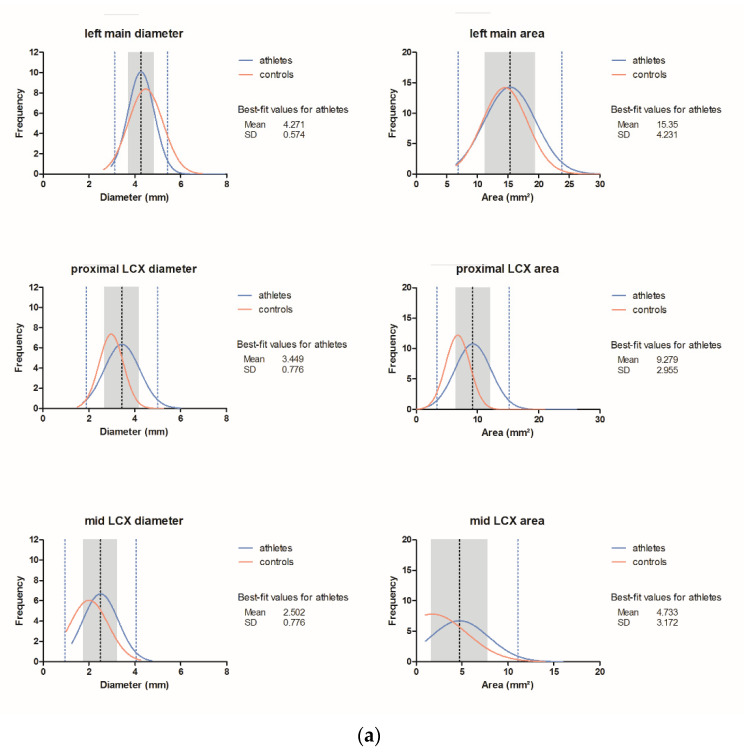

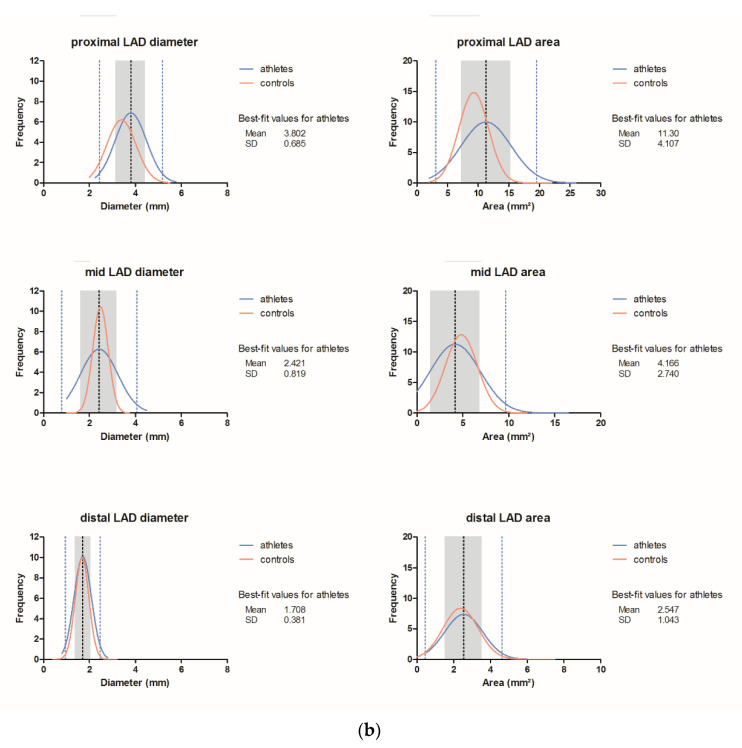

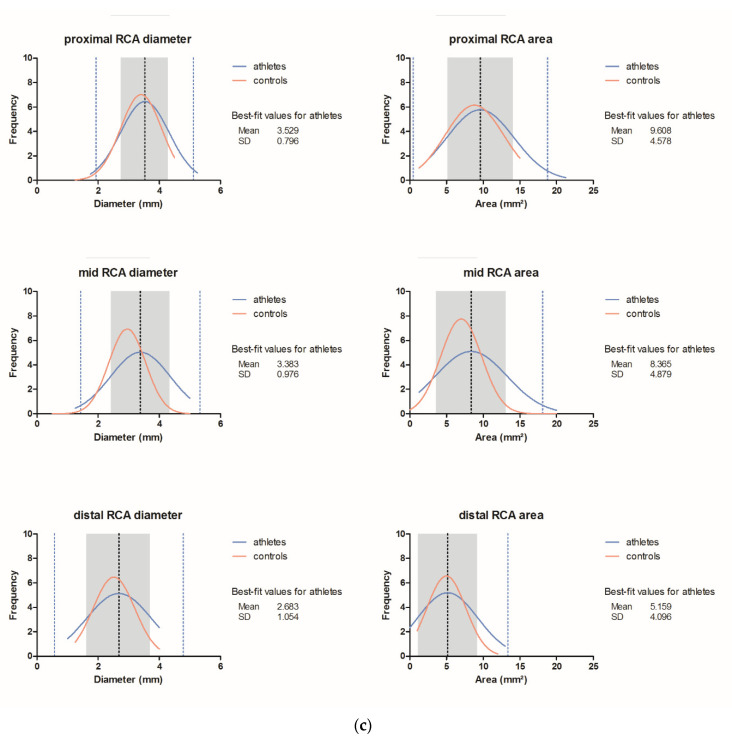
Coronary vessel dimensions (mean diameter and area): normal values for athletes and controls. Dashed lines ± 2 SD (lower and upper border) and ± 1 SD (inner grey zone)**;** blue line = athletes; red line = controls; ---- best fit value. (**a**) Left main (LM) and circumflex (CX) artery; (**b**) Left anterior descending (LAD); (**c**) Right coronary artery (RCA).

Three-dimensional vessel volumes (Figure 1b) correlated moderately with the proximal mean diameter and area of the RCA (r = 0.442 and r = 0.45, *p* < 0.001, Pearson) and the LAD (r = 0.438 for both, *p* < 0.001, Spearman). Figure 2 shows histograms with ± 1 SD and 2 SD for all coronary segments.

## 4. Discussion

Our study shows that coronary vessel dimensions (mean diameter and area) for proximal LAD, proximal and mid CX and RCA were significantly larger in endurance athletes than in sedentary controls. Left main and all distal segments were slightly wider but not statistically different. Known adaptations to high training volumes in endurance sports include an increase in capillaries in muscle fibers, the growth and increase in contractility of cardiomyocytes, mediated by a complex interaction of all growth factors [2]. An increase in vasodilation capacity is another known phenomenon [4]. Whether or not arterial remodeling occurs has recently sparked a debate among scientists. The “*athlete’s artery*” may be characterized by increased wall thickness and enlarged arteries [3]. However, there is only limited and anecdotal data about vessel size in endurance athletes, with very small size samples and mainly targeting adenosine triggered vasodilation capacity: *Haskell* et al. reported in 1983 that 11 ultra-distance runners had an increased vasodilation capacity of the proximal LAD after nitroglycerin administration, as compared to inactive controls, while there was no difference at rest. Invasive angiography was used, which is prone to inaccuracies due to 2D projection and distortion effects. Kozakova et al. (2007) found that LM area was enlarged in 15 athletes and increased along with coronary flow reserve (CFR) measured by invasive angiography [5] and transesophageal echocardiography. Similarly, Windecker el al (2001) utilized invasive angiography and echocardiography to study eight volunteers before and after an intensive sustained exercise program for 9 months, after which vasodilative capacity improved markedly, and vessels showed structural remodeling [6]. Other studies have found enlarged arteries in the lower limbs of cyclists or forearms of rowers [3] by ultrasonography, but no study yet has applied coronary CTA for exact sizing of coronary vessel dimensions. We used quantitative coronary CTA, uniquely, which is the most accurate tool for quantification of vessel lumen diameters and area due to its high resolution and the capability of real 3D measurements without distortion due to 2D projection by invasive coronary angiography. Our study shows that both vessel diameters and the area were enlarged in athletes. Total vessel volumes were also higher, though did not reach a significant level. This may be explained because distal segments with >2 mm were included from volume analysis, which were not different in terms of diameter and area dimensions but represent a high percentage of total vessel volume. Beyond this, the spatial resolution of CT in distal segments may be not high enough to reveal differences. In line, falsely reduced computed tomography fractional flow reserve (CT-FFR) values in distal segments have been previously observed in athletes [13]. The correlation of 3D vessel volumes and with quantitative 2D sizing parameters such as the mean diameter and area by CTA was only moderate, which is explained by the fact that 2D vessel size and 3D volume measurements cannot produce equal results due to their distinct geometry. 2D vessel size (area and diameters) was measured at one (largest) image slice of the target segment only, while vessel volume represents the entire vessel volume.

Our study provides, for the first time, evidence that endurance athletes exhibit enlarged coronary arteries at rest. The differential diagnosis of “Athletes’ arteries” (Figure 1a) from normal, Kawasaki disease [14] and multisystemic inflammation syndrome [8] must be made carefully by performing a clinical work-up including specific blood sample sets (c-reactive protein, blood sedimentation rate, etc.) as recommended [8,13]. Rheumatic, autoimmune or other systemic diseases triggering vasculitis or HIV may also involve coronary arteries and result in vessel wall inflammation [15] and focal or diffuse coronary ectasia. Patients with a history or diagnosis of these conditions were therefore excluded from this study. Furthermore, coronary CTA offers the advantage of a simultaneous evaluation of coronary anomalies, cardiac function and occult coronary artery disease, as well as congenital heart disease [5]. Coronary CTA is indicated, for example, in athletes with new onset of arrhythmia or chest pain [16].

Finally, of note, ectatic coronary segments may also occur during Kawasaki disease, multisystemic inflammation syndrome after COVID-19, the ectatic form of atherosclerosis and other types of vasculitis. Therefore, these diseases must be distinguished from “athlete’s arteries”.

### Study Limitations

Study design was retrospective with an inherent bias. Body mass index was slightly lower in athletes. However, there was no difference in the body surface area (BSA). The prevalence of smoking was slightly higher in controls.

## 5. Conclusions

Endurance athletes exhibit larger proximal LAD, proximal/mid CX and RCA segments (“athletes’ arteries”). Our data may be useful in clinical practice to distinguish normal vessel enlargement in endurance athletes from normal.

## Figures and Tables

**Table 1:** Study population.

	Athletes *n* = 51	Controls *n* = 47	*p*-Value
Age (years)	54.5 ± 11	57.7 ± 13	0.187
Females	11 (38%)	18 (22%)	0.71
BMI kg/m^2^	24.1 ± 3.4	26.3 ± 3.4	<0.001 *
BSA m^2^	1.89 ± 0.17	1.94 ± 0.21	0.220
Art. hypertension	15 (29%)	22 (47%)	0.077
Smoking	8 (4%)	13 (26%)	0.151
Positive fam. history	18 (35%)	22 (47%)	0.459
Dyslipidemia	20 (39%)	21 (45%)	0.588
Diabetes	0 (0%)	4 (9%)	0.098
Stenosis > 50%	8 (15.7%)	13 (27.7%)	0.151
CACS (AU)	32.3 ± 80	109.1 ± 210	0.067

Abb. Parametric data are presented as mean ± SD, counts as N (%). CACS = coronary artery calcium score, AU = Agatston units, BMI = body mass index (kg/m^2^), BSA = Body surface area (BSA). * 2-tailed *p*-value, significant.

**Table 2:** Coronary artery size by CTA: athletes vs. controls (*n* = 98).

		Athletes *n* = 51	Controls *n* = 47	*p*-Value
DIAMETER (mm) (mean **)				
RCA * Prox mid distal		3.68 ± 0.72 3.56 ± 0.77 2.72 ± 0.67	3.43 ± 0.56 3.19 ± 0.67 2.63 ± 0.59	0.082 0.028 *** 0.428
LM		4.6 ± 0.95	4.5 ± 0.81	0.809
LAD Prox mid distal		3.84 ± 0.67 2.53 ± 0.76 1.74± 0.38	3.54 ± 0.67 2.41 ± 0.64 1.64 ± 0.52	0.019 *** 0.628 0.253
CX Prox mid distal		3.46 ± 0.77 2.59 ± 0.74 1.97 ± 0.60	3.07 ± 0.77 2.17 ± 0.73 1.62 ± 0.61	0.018 *** 0.008 *** 0.315
AREA (mm^2^)				
RCA * Prox mid distal		10.94 ± 4.12 10.28 ± 4.38 6.14 ±2.86	9.35 ± 3.00 8.20 ±3.44 5.65 ±2.5	0.048 *** 0.026 *** 0.463
LM		17.3 ± 7.4	16.36 ± 6.23	0.708
LAD Prox mid distal		11.91 ± 4.13 5.45 ± 3.24 2.43 ± 1.05	10.17 ± 3.99 4.77 ± 2.33 2.21 ± 1.33	0.019 *** 0.621 0.226
CX Prox mid distal		9.73 ± 4.34 5.59 ± 3.16 3.32 ± 2.09	7.83 ± 4.89 4.07 ± 2.78 2.26 ± 1.74	0.026 *** 0.009 *** 0.315
Vessel Volume and Myocardial Mass				
RCA	mm^3^	1082.61 ± 468.75	999.32 ± 368.80	0.434
LAD	mm^3^	695.09 ± 214.25	689.14 ± 295.29	0.924
CX	mm^3^	622.70 ± 427.52	516.56 ± 414.2	0.129
M-Mass	mg	134.21 ± 28.08	122.74 ± 34.34	0.047 ***

Abbr. RCA * only right-dominant and intermediate supply type right coronary arteries (RCA) were included: *n* = 41 vs. *n* = 42. ** the mean of 2 diameter (±1 SD) is reported; *** *p* < 0.05; M-Mass = myocardial mass.

## Data Availability

The data have not been made publicly available. Data are stored locally and can be provided upon request.

## Abbreviations

CTA = computed tomography angiography. LAD = left anterior descending. CX = circumflex artery. RCA = right coronary artery. LM = left main. AHA = American Heart Association. ECG = electrocardiogram. HU = Hounsfield units.
